# Development and validation of a multimodal nomogram predicting anxiety and depression in Parkinson’s disease: integrating plasma biomarkers and clinical phenotypes

**DOI:** 10.3389/fnagi.2026.1896303

**Published:** 2026-08-03

**Authors:** Guidong Liu, Yanqin Geng, Hanwen Zhang, Ping Ding, Yiping Zhou, Yiqing Ren, Wenshi Wei

**Affiliations:** 1 Department of Neurology, Huadong Hospital Affiliated to Fudan University, Shanghai, China; 2 Department of Gerontology, Shanghai Key Laboratory of Clinical Geriatric Medicine, Huadong Hospital Affiliated to Fudan University, Shanghai, China

**Keywords:** Parkinson’s disease, anxiety, depression, neurofilament light chain (NfL), nomogram, clinical prediction model, non-motor symptoms

## Abstract

**Introduction:**

Anxiety and depression are prevalent, disabling, yet frequently underdiagnosed non-motor symptoms in Parkinson’s disease (PD). This study aimed to develop and validate a non-invasive model predicting these affective disorders by integrating peripheral blood biomarkers with standardized clinical scales to facilitate early screening.

**Methods:**

We retrospectively analyzed data from 290 patients with PD, who were randomly allocated into a training cohort (*n =* 203) and a validation cohort (*n =* 87). Baseline plasma neurofilament light chain (NfL) levels and clinical phenotypes were assessed. Independent risk factors were determined via multivariate logistic regression analysis to construct the clinical prediction nomogram. Model performance was comprehensively evaluated using the area under the receiver operating characteristic curve (AUC) for discrimination, calibration curves for risk consistency, and decision curve analysis (DCA) for clinical utility.

**Results:**

Multivariate logistic regression identified plasma NfL, Hoehn and Yahr stage, MMSE score, and MDS-UPDRS Part III score as independent predictors for anxiety and depression in PD patients (all *p* < 0.05). The established model exhibited high discriminative power, achieving an AUC of 0.94 (95% CI: 0.873–0.964) in the training cohort and 0.84 (95% CI: 0.782–0.879) in the validation cohort. Calibration curves demonstrated excellent consistency between predicted and actual probabilities, and DCA confirmed strong clinical net benefits.

**Discussion:**

In conclusion, combining peripheral plasma NfL levels with standard clinical phenotypes provides an objective, quantifiable, and non-invasive tool for early risk stratification of affective disorders in PD. This multimodal nomogram effectively expands the therapeutic window for timely personalized psychiatric interventions, potentially improving long-term quality of life and clinical outcomes for PD patients.

## 1 Introduction

Parkinson’s disease (PD) is widely recognized as a heterogeneous multisystem neurodegenerative disorder characterized not only by classic motor features but also by a broad spectrum of non-motor symptoms (NMS). Notably, certain NMS, such as olfactory dysfunction, autonomic dysregulation, and REM sleep behavior disorder, frequently precede the onset of motor manifestations by years or even decades. As the disease progresses, these symptoms often overlap, creating a complex clinical phenotype driven by shared neuroanatomical and pathophysiological pathways. Within this extensive NMS spectrum, neuropsychiatric disturbances—predominantly anxiety and depression—co-occur frequently with cognitive impairment, sleep disorders, and prominent fatigue, compounding the clinical burden and drastically reducing the quality of life for PD patients (Leite Silva et al., 2023). Therefore, characterizing the specific risk profiles of anxiety and depression within this interconnected multisystem framework is of paramount clinical importance. These affective disorders affect approximately 35–50% of patients with PD, frequently presenting as an early “neuropsychiatric triad” that drastically compromises quality of life (Gibson et al., 2022). Despite their clinical significance, anxiety and depression in PD remain chronically underdiagnosed and undertreated (Sujith et al., 2023). This diagnostic dilemma stems primarily from symptom attribution bias; somatic manifestations such as fatigue, sleep disturbances, and psychomotor changes present a heavy phenotypic overlap, being confounded either as core motor features of PD or as traditional vegetative symptoms of primary psychiatric disorders (Sujith et al., 2023). Currently, clinical screening relies almost exclusively on subjective neuropsychological scales. These toolsets are highly susceptible to investigator bias and, more importantly, lack the capacity to reflect the underlying microstructural and molecular neurodegeneration prior to macroscopic clinical deterioration. Therefore, there is an urgent need to establish objective, quantifiable, and minimally invasive biomarkers to optimize early risk stratification and personalize psychiatric interventions. The complex, heterogeneous pathophysiology of psychiatric complications in PD involves multi-dimensional pathways, including central axonal degeneration, neuroinflammation, and protein co-pathology, which can be captured via peripheral liquid biopsies. Neurofilament light chain (NfL) has emerged as a sensitive blood-based indicator of subclinical axonal injury, with elevated levels tracking closely with affective symptom severity in neurodegenerative cohorts (Yin et al., 2022). Concurrently, glial activation and systemic neuroinflammation—marked by alterations in glial fibrillary acidic protein (GFAP)—disrupt the functional connectivity within the striatal-thalamic-cortical and limbic networks, directly driving severe anhedonia (Wang et al., 2025). Furthermore, the cross-talk between classic neurodegenerative pathologies, such as phosphorylated tau (p-tau) and amyloid-beta (A*β*1–42), accelerates cognitive-affective network degradation, creating a synergistic pathological cascade that amplifies neuropsychiatric vulnerability (Liu et al., 2015). While these fluid biomarkers offer critical insights into central molecular dynamics, single-marker approaches often yield insufficient predictive accuracy due to the highly individual variations in PD phenotypes. Integrating these multidimensional peripheral biomarkers with key clinical scales represents a promising strategy to overcome these individual variations. To achieve this, predictive nomograms combining blood-based biomarkers with distinct clinical phenotypes (such as disease stage and cognitive scores) provide an accessible, high-compliance framework for real-world screening. To date, however, few validated models have successfully bridged ultra-sensitive plasma biomarker panels with routine clinical metrics to predict the specific risk of concurrent anxiety and depression in patients with PD. To address this gap, this study aims to construct and validate a novel multimodal risk prediction model using a longitudinal, well-characterized PD cohort. By evaluating baseline plasma levels of NfL, GFAP, p-tau, and A*β*1–42 alongside standardized clinical assessments (including H&Y stage, MMSE, and MDS-UPDRS scores) (Hoehn & Yahr, 1967; Goetz et al., 2008), we seek to deliver a robust, non-invasive early-warning system. Ultimately, this framework is intended to advance the therapeutic window for neuropsychiatric complications, guide preemptive individualized treatment, and help unravel the “peripheral-central” interactions underlying affective symptoms in PD from a translational perspective.

## 2 Materials and methods

### 2.1 Study population

We retrospectively screened 290 patients with Parkinson’s disease (PD) admitted to our hospital between January 2016 and January 2024. All patients provided written informed consent. The inclusion criteria were: (1) diagnosed with PD by at least two specialists according to the 2016 Chinese and 2015 MDS clinical diagnostic criteria (Postuma et al., 2015); (2) complete clinical and follow-up data; (3) disease duration <5 years. Exclusion criteria included: (1) secondary or atypical parkinsonism; (2) hepatic or renal insufficiency; (3) other co-existing cognitive disorders (e.g., Alzheimer’s disease, dementia with Lewy bodies); (4) neurosurgical treatment within the past 3 months; (5) active malignancies or autoimmune diseases. The overall cohort was randomly allocated into a training cohort (*n =* 203) and a validation cohort (*n =* 87) at a 7:3 ratio.

### 2.2 Clinical and neuropsychological assessments

Demographic and clinical baseline data were collected. Motor symptoms were assessed using the MDS-UPDRS Part III and Hoehn and Yahr (H&Y) staging in the medication “OFF” state (after a ≥ 12-h withdrawal of anti-PD medications). Global non-motor symptoms and cognitive functions were evaluated via the Non-Motor Symptoms Scale (NMSS) and the Mini-Mental State Examination (MMSE), respectively. Depression and anxiety were tracked using the 17-item Hamilton Depression Rating Scale (HAMD-17) and the Hamilton Anxiety Rating Scale (HAMA). Patients were classified into the Anxiety/Depression group if they presented with a score ≥ 7 on either scale at any follow-up visit, or initiated anxiolytic/antidepressant treatment during follow-up. The remaining patients who consistently maintained scores < 7 and required no psychiatric medications were defined as the Non-Anxiety/Depression Group. In the training cohort, 81 patients were categorized into Anxiety/Depression group and 122 into the Non-Anxiety/Depression Group. we analyzed the cross-sectional prevalence and associated biomarker correlation within the patient cohort.

### 2.3 Blood sampling and ultra-sensitive biomarker quantification

Fasting peripheral venous blood (5 mL) was collected between 8:00 and 9:00 a.m. into EDTA tubes following a ≥ 12-h anti-PD medication washout. Samples were centrifuged at 2,000 × g for 15 min at 4 °C within 1 h of collection. Plasma supernatants were aliquoted (100 μL/tube) and stored at −80 °C, undergoing a maximum of one freeze–thaw cycle prior to batch analysis.

Plasma concentrations of NfL, p-tau, GFAP, A*β*1–42, and A*β*40 were quantified in duplicate on Single Molecule Array (Simoa) technology on the automated Simoa HD-1 Analyzer™ platform (Quanterix, Billerica, MA, USA) using the Simoa NF-light Reagent Kit (Quanterix, Cat#103186) and Simoa Neuro 3-Plex A Kit (Quanterix, Cat# 101995) according to the manufacturer’s instructions. Following the manufacturer’s protocols. Quality control criteria required an intra-assay coefficient of variation (CV) < 10% (samples with CV > 15% were re-tested) and an inter-assay CV < 15%. All laboratory personnel were completely blinded to the patients’ clinical metadata and longitudinal outcomes by using sequentially numbered experimental IDs.

### 2.4 Statistical analysis

Statistical analyses were performed using SPSS 27.0 (IBM Corp., Armonk, NY, USA) and R software. Data normality was evaluated via the Shapiro–Wilk test. Normally distributed continuous variables are expressed as mean ± standard deviation and compared using the independent-sample *t*-test; non-normally distributed continuous data are presented as medians with interquartile ranges and analyzed via the Mann–Whitney *U* test. Categorical variables are expressed as frequencies (percentages) and compared using the *χ*^2^ test or Fisher’s exact test.

Missing clinical data (missing rate < 10%, assumed missing at random) were handled using multiple imputation by chained equations (MICE, 5 iterations) in R. Plasma biomarker concentrations falling below the lower limit of quantification (LLOQ) were imputed using the LLOQ/ √2 method.

Univariate and multivariate binary logistic regression analyses were conducted to identify independent risk factors for concurrent anxiety and depression. Model construction strictly adhered to the TRIPOD statement and the 10 events per variable (EPV) criterion to prevent overfitting. The predictive performance and clinical utility of the developed nomogram were comprehensively validated using the area under the receiver operating characteristic curve (AUC), calibration curves, and decision curve analysis (DCA). Statistical significance was defined as two-tailed *p* < 0.05.

### 2.5 Ethical approval

This study was approved by the Ethics Committee of Huadong Hospital Affiliated to Fudan University (Approval No.: 2019 K011). Written informed consent was obtained from all participants prior to enrollment.

## 3 Results

### 3.1 Baseline characteristics

The baseline demographics and clinical characteristics of the study population are summarized in Table 1. The mean age was 69 years in the training cohort and 68 years in the validation cohort, with males accounting for 50.25 and 39.08% of the patients, respectively. No statistically significant differences were observed between the two cohorts regarding age, sex, BMI, residence, disease duration, levodopa equivalent daily dose (LEDD), prevalence of hypertension and diabetes, educational level, or marital status (all *p* > 0.05), establishing that the cohorts were well-balanced and comparable.

**Table 1:** Comparison of general information of patients in the training cohort and the validation cohort.

General demographic data		Training cohort (*n =* 203)	Validation cohort (*n =* 87)	Z/*χ*^2^ value	*P*-value
Age (years)		69.00 (65.00, 74.00)	68.00 (64.00, 74.00)	−0.371	0.711
Sex *n* (%)	Male	102 (50.25)	34 (39.08)	3.049	0.081
	Female	101 (49.75)	53 (60.92)		
BMI (kg/m^2^)		22.74 (20.13, 25.55)	22.88 (20.50, 25.51)	−0.753	0.451
Place of residence	Urban	150 (73.89)	17 (19.54)	1.435	0.231
	Rural	53 (26.11)	70 (80.46)		
Course of disease (years)		5.00 (3.00, 7.00)	5.00 (2.00, 8.00)	−0.177	0.860
LEDD (mg/d)		493.06 (393.55, 581.76)	500.07 (384.68, 591.76)	−0.057	0.954
Hypertension (*n*, %)	Yes	76 (37.44)	31 (35.63)	0.085	0.770
	No	127 (62.56)	56 (64.37)		
Diabetes (*n*, %)	Yes	45 (22.17)	21 (24.14)	0.135	0.714
	No	158 (77.83)	66 (75.86)		
Education *n* (%)	Less than college education	139 (68.47)	63 (72.41)	0.447	0.504
	College or above	64 (31.53)	24 (27.59)		
Marital status	Married	104 (51.23)	55 (63.22)	3.533	0.060
	Others	99 (48.77)	32 (36.78)		
H&Y stage		2.00 (2.00, 3.00)	3.00 (2.00, 3.00)	−0.772	0.44
MMSE score		26.00 (25.00, 28.00)	25.00 (24.00, 27.00)	−1.451	0.147
MDS-UPDRS Part III score		32.00 (28.00, 35.00)	31.00 (25.00, 35.00)	−1.554	0.120
GFAP (pg/ml)		70.57 (56.28, 82.90)	75.24 (59.00, 83.65)	−0.992	0.321
NfL (pg/ml)		12.15 (10.26, 14.66)	12.59 (10.29, 15.09)	−0.996	0.319
p-tau (pg/ml)		1.62 (1.23, 1.94)	1.40 (1.14, 1.85)	−1.516	0.130
A*β*1-42 (pg/ml)		7.51 (6.48, 8.25)	7.31 (6.28, 8.05)	−1.203	0.229
A*β*40 (pg/ml)		85.23 (74.08, 98.78)	87.32 (77.54, 101.13)	−1.056	0.291

### 3.2 Univariate analysis and feature selection

Univariate Analysis: In the training cohort, the H&Y stage was significantly higher in the Anxiety/Depression group than in the non-Anxiety/Depression group (*p* < 0.001), suggesting that patients with greater disease severity are more susceptible to anxiety and depression. Concurrently, the MMSE score was significantly decreased in the Anxiety/Depression group (p < 0.001), indicating that cognitive impairment could be a critical predisposing factor for anxiety and depression. Furthermore, the MDS-UPDRS Part III score, along with plasma GFAP, p-tau, and NfL levels, were all significantly elevated in the Anxiety/Depression group (all *p* < 0.05). In contrast, the comparison of marital status and plasma A*β*1-42 yielded borderline significant *p*-values, *p* < 0.1; these findings are detailed in Table 2.

**Table 2:** Univariate predictors of anxiety and depression in PD patients.

General demographic data		Anxiety/depression group (*n =* 81)	Non- anxiety/depression group (*n =* 122)	Z/*χ*^2^ value	*P*-value
Age (years)		68.00 (65.00, 73.00)	69.50 (65.00, 74.25)	−0.759	0.448
Sex *n* (%)	Male	46 (56.79)	56 (45.90)	2.309	0.129
	Female	35 (43.21)	66 (54.10)		
BMI (kg/m^2^)		22.51 (20.09, 25.58)	22.83 (20.17, 25.11)	−0.066	0.947
Place of residence	Urban	61 (75.31)	89 (72.95)	0.140	0.708
	Rural	20 (24.69)	33 (27.05)		
Course of disease (years)		5.00 (3.00, 8.00)	5.00 (3.00, 7.00)	−0.124	0.901
LEDD(mg/d)		511.32 (423.46, 575.76)	483.87 (383.41, 586.99)	−0.729	0.466
Hypertension (*n*, %)	Yes	36 (44.44)	40 (32.79)	2.824	0.093
	No	45 (55.56)	82 (67.21)		
Diabetes (*n*, %)	Yes	18 (22.22)	27 (22.13)	0.000	0.988
	No	63 (77.78)	95 (77.87)		
Education *n* (%)	Less than college education	56 (69.14)	83 (68.03)	0.027	0.868
	College or above	25 (30.86)	39 (31.97)		
Marital status	Married	35 (43.21)	69 (56.56)	3.471	0.062
	Others	46 (56.79)	53 (43.44)		
H&Y stage		3.00 (2.00, 4.00)	2.00 (2.00, 3.00)	−6.292	<0.001
MMSE score		23.00 (21.00, 25.00)	27.00 (26.00, 29.00)	−10.079	<0.001
MDS-UPDRS Part III score		34.00 (31.00, 38.00)	29.00 (25.00, 35.00)	−5.855	<0.001
GFAP (pg/ml)		76.82 (59.78, 85.71)	65.45 (52.82, 80.02)	−2.834	0.005
NfL (pg/ml)		13.81 (12.13, 16.02)	11.02 (9.23, 13.43)	−6.168	<0.001
p-tau (pg/ml)		1.87 (1.34, 2.09)	1.52 (1.17, 1.82)	−3.784	<0.001
A*β*1-42 (pg/ml)		7.41 (6.20, 8.09)	7.58 (6.55, 8.43)	−1.857	0.063
A*β*40 (pg/ml)		84.71 (74.26, 99.62)	85.40 (74.00, 97.78)	−0.084	0.933

### 3.3 Multivariate logistic regression analysis for incident affective symptoms

**Figure 1:**
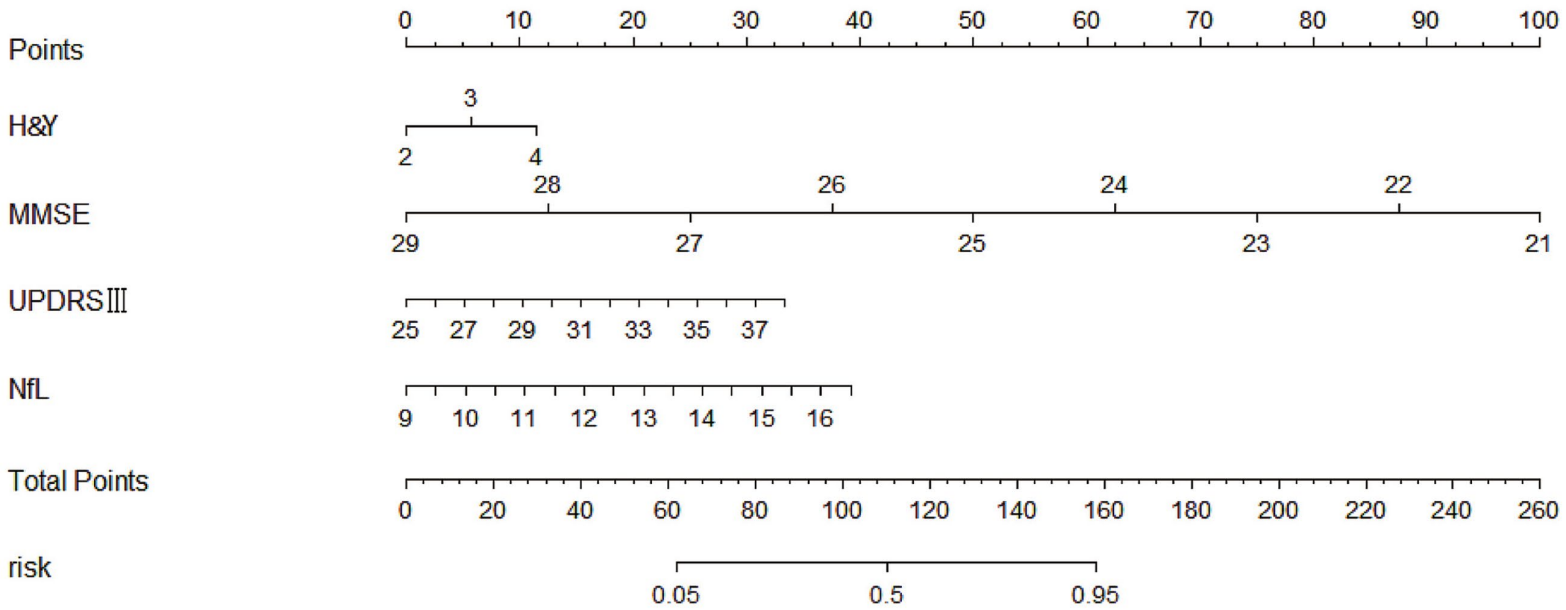
Predictive nomogram model.

Candidate variables demonstrating statistical significance (*p* < 0.05) or a strong statistical trend (*p* < 0.10, specifically marital status and plasma A*β*1-42) in the univariate analysis were simultaneously introduced into a multivariate binary logistic regression model using the forced entry method (Enter method). This approach ensures that all potential baseline confounders were completely controlled for simultaneously, allowing for the rigorous identification of true independent risk/protective factors for incident anxiety and depression (0 = No, 1 = Yes) (Figure 1).

After fully adjusting for other concurrent candidate variables (including plasma p-tau, A*β*1–42, GFAP, and marital status), plasma NfL, H&Y stage, and MDS-UPDRS Part III score remained as significant independent risk factors, plasma NfL (OR = 1.503, 95% CI: 1.142–1.978, *p* = 0.004), H&Y stage (OR = 2.377, 95% CI: 1.175–4.807, *p* = 0.016), and MDS-UPDRS Part III score (OR = 1.321, 95% CI: 1.120–1.557, *p* = 0.001) were identified as independent risk factors for incident anxiety and depression (Table 3). Conversely, MMSE score served as a significant independent protective factor (OR = 0.207, 95% CI: 0.110–0.390, *p* < 0.001). Other variables, including plasma p-tau (*p* = 0.223), A*β*1–42 (*p* = 0.094), GFAP (*p* = 0.899), and marital status (*p* = 0.621), did not retain statistical significance in the multivariate model. These findings are detailed in Table 3.

**Table 3:** Results of binary logistic regression analysis.

Dependent variable	B	B (SE)	Wald	*P*	Exp (B)	95% CI
NfL	0.408	0.140	8.460	0.004	1.503	1.142–1.978
P-tau	0.837	0.688	1.482	0.223	2.310	0.600–8.896
A*β*1-42	−0.436	0.260	2.812	0.094	0.646	0.388–1.076
H&Y stage	0.866	0.359	5.803	0.016	2.377	1.175–4.807
MMSE score	−1.573	0.322	23.829	0.000	0.207	0.110–0.390
MDS-UPDRS Part III score	0.278	0.084	10.987	0.001	1.321	1.120–1.557
GFAP	−0.002	0.017	0.016	0.899	0.998	0.965–1.032
Marital status (1)	0.316	0.640	0.244	0.621	1.372	0.391–4.807
Constant	24.903	7.559	10.854	0.001	6.536	–

### 3.4 Nomogram construction, calibration, and predictive performance

Based on the multivariate analysis, plasma NfL, H&Y stage, MMSE score, and MDS-UPDRS Part III score were integrated to construct the predictive nomogram, expressed as follows:


$$\begin{array}{l} \text{Logit}(P)=24.903+0.408\times \mathrm{NfL}+0.866\times H\&Y\\ -1.573\times \text{MMSE}+0.278\times \mathrm{MDS}-\text{UPDRS Part}\;\mathrm{III} \end{array}$$

**Figure 2:**
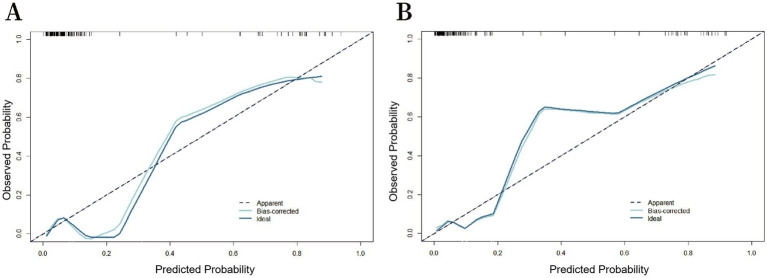
Calibration curves of the nomogram for predicting affective disorders (anxiety and depression) in patients with Parkinson’s disease. **(A)** Calibration curve in the training cohort (*n* = 203), showing excellent agreement between the predicted probability of affective disorders (x-axis) and the actual observed risk (y-axis). **(B)** Calibration curve in the validation cohort (*n* = 87), demonstrating the robust consistency and generalizability of the prediction model.

Where P represents the predicted probability of incident anxiety or depression. Calibration curves demonstrated excellent consistency between predicted and observed risks, with slope values approaching 1 in both the training and validation cohorts (Figures 2A,B).

**Figure 3:**
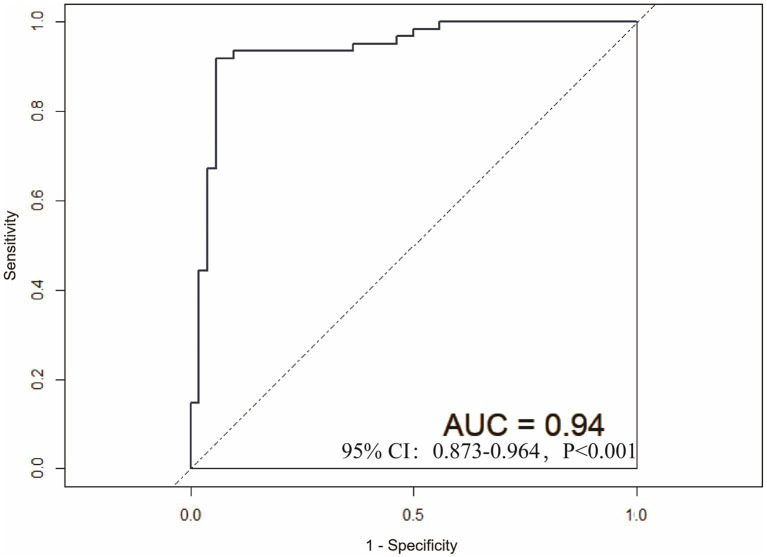
ROC curve of the model in the training cohort.

**Figure 4:**
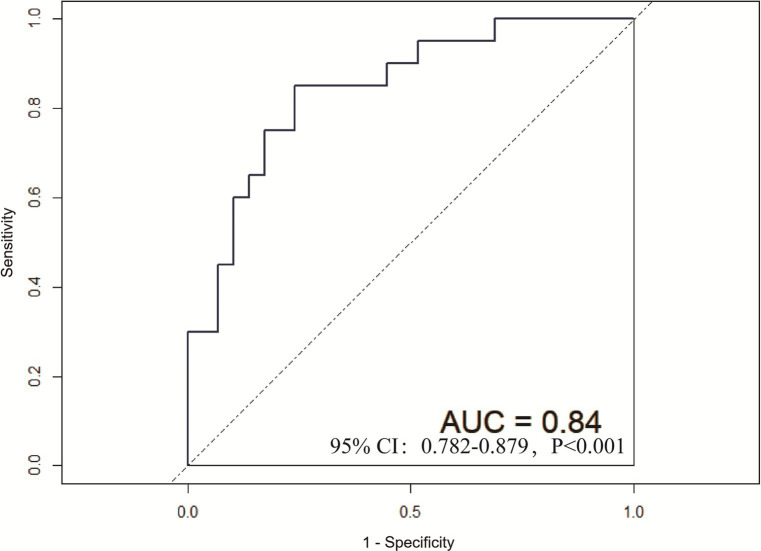
ROC curve of the model in the validation cohort.

Receiver operating characteristic (ROC) analysis confirmed robust discriminative performance (Figures 3, 4). In the training cohort, the area under the curve (AUC) was 0.94 (standard error [SE] = 0.036, 95% CI: 0.873–0.964); at the optimal Youden index of 0.91, the model yielded a sensitivity of 94.8% and a specificity of 96.6%. In the validation cohort, the AUC was 0.84 (SE = 0.072, 95% CI: 0.782–0.879), with a sensitivity of 85.4% and a specificity of 77.5% at the optimal Youden index of 0.63.

### 3.5 Decision curve analysis

**Figure 5:**
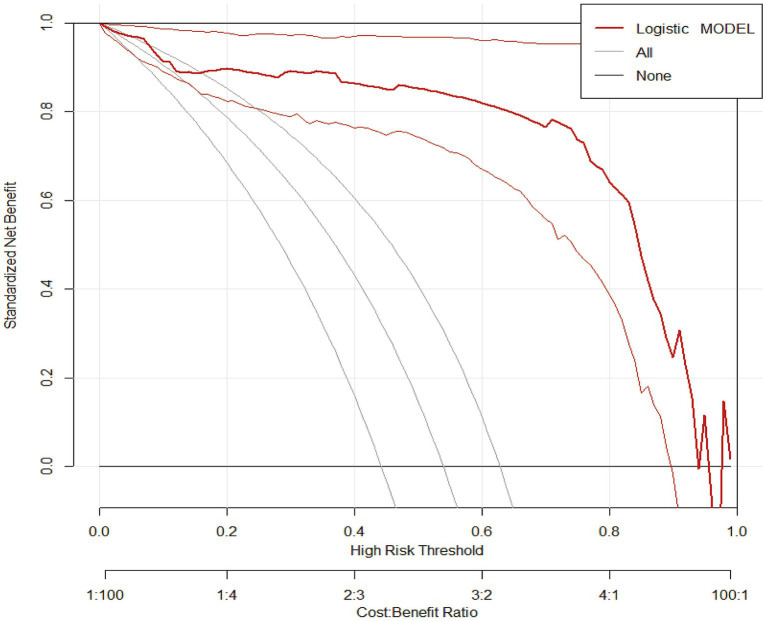
Decision curve analysis (DCA) of the predictive model for concurrent affective disorders in Parkinson’s disease.

Decision curve analysis (DCA) was performed to evaluate the clinical utility of the model (Figure 5). In both the training and validation cohorts, the model’s net benefit curve remained significantly higher than both extreme strategies (“intervene all” and “intervene none”) across a wide, clinically relevant threshold probability range of 5 to 85%.

## 4 Discussion

Parkinson’s disease (PD) is a progressive neurodegenerative disorder marked not only by motor symptoms but also by critical non-motor manifestations, particularly anxiety and depression, which severely impair patients’ quality of life (Ahmad et al., 2023). Recently, plasma biomarkers have garnered substantial attention for their potential roles in disease diagnosis and prognostic evaluation (Gan et al., 2025). Accumulating evidence indicates a profound pathological synergy between *α*-synuclein and traditional Alzheimer’s disease hallmarks A*β*1–42 and tau. A*β*1–42 oligomers and hyperphosphorylated tau can cross-seed α-synuclein, promoting its neocortical propagation into the limbic system. This co-pathology triggers widespread microglial activation and structural disconnection within frontostriatal networks, thereby predisposing PD patients to a combined phenotype of cognitive deterioration and affective disorders. This study sought to develop and validate a multi-index nomogram model, integrating clinical phenotypes and peripheral biomarkers, to predict affective disorders in a retrospective cohort of PD patients, thereby providing a more precise empirical foundation for targeted clinical interventions.

In univariate analyses, variables including NfL, p-tau, A*β*1–42, H&Y stage, MMSE score, MDS-UPDRS III score, GFAP, and marital status showed significant associations with affective disorders. Subsequent multivariate regression analysis identified H&Y stage, MMSE score, MDS-UPDRS III score, and plasma NfL as robust, independent predictors. Specifically, the H&Y stage directly reflects disease staging and daily disability; its elevation in the affective disorder group highlights that advanced neurodegeneration predisposes patients to severe psychological distress, driven by diminished daily functioning, social withdrawal, and compromised self-esteem (Elcadi et al., 2025). Similarly, decreased MMSE scores underscored the intricate link between cognitive impairment and affective symptoms, wherein cognitive decline reduces a patient’s capacity to cope with illness-related challenges, fostering feelings of helplessness (Sagna et al., 2014). Furthermore, elevated MDS-UPDRS Part III score underscored that motor dysfunction and symptom fluctuations introduce profound psychological stress and helplessness, amplifying vulnerability to anxiety and depression (Cong et al., 2022; Kwok et al., 2025).

Concurrently, peripheral biomarkers played an indispensable role in our model. Plasma NfL is an established, ultra-sensitive index of axonal injury and neurodegeneration (Halliday & McCann, 2010). Our finding that NfL levels were significantly elevated in the affective disorder group suggests that structural neuronal damage may disrupt neurotransmitter systems essential for mood regulation, potentially mediated by underlying neuroinflammatory cascades (Quadalti et al., 2021). Serial monitoring of plasma NfL offers scientific utility in tracking disease progression, consistent with the findings reported by Gibson et al. (Gibson et al., 2022). In this study, the robust predictive capacity of our final multivariable framework underscores the intricate interplay between neuropsychiatric states and other dimensions of the NMS spectrum. Specifically, the strong protective effect of the MMSE score directly reflects how cognitive decline and dementia share cross-cutting neural substrates with affective disorders in PD, such as the progressive degeneration of ascending monoaminergic (serotonergic, noradrenergic) and cholinergic pathways. Furthermore, elevated plasma NfL levels reflect cumulative axonal damage that is not confined to motor tracts but spans across diffuse non-motor networks. This axonal degeneration likely disrupts structural connectivity within frontostriatal and limbic networks, driving a cascade that simultaneously triggers anxiety, depression, fatigue, and autonomic dysfunction. By situating emotional distress within this broader, interconnected NMS architecture rather than viewing it as an isolated psychological reaction, our findings provide a more holistic biomarker-phenotype linkage for personalized clinical management.

Intriguingly, although plasma GFAP, p-tau, and A*β*1–42 are closely linked to PD pathology, they lost statistical significance in our multivariate model (*p* > 0.05). This phenomenon is likely attributable to multicollinearity and overlapping pathological mechanisms. As a broad downstream marker of axonal injury, plasma NfL may integrate the cumulative damage driven by neuroinflammation (GFAP-related) and tau pathology (p-tau-related), thereby exhibiting superior independent predictive power. Additionally, A*β*1–42 shared inherent pathological collinearity with the MMSE score, which reflects cortical cognitive reserve. Given the formidable statistical explanatory power of the MMSE score in our model (Wald = 23.829), the predictive value of A*β*1–42 was likely largely accounted for or masked by this phenotypic variable. This highlights that combining downstream core biomarkers like NfL with multi-dimensional clinical scales offers greater translational value for risk stratification than relying solely on upstream, localized pathology markers.

The developed nomogram demonstrated robust predictive performance, clinical utility, and favorable discrimination and calibration. ROC analysis revealed high areas under the curve (AUC) in both the training and validation cohorts, indicating an optimal balance of sensitivity and specificity. The calibration curves demonstrated high concordance between predicted risks and actual outcomes. Furthermore, decision curve analysis (DCA) indicated a substantial net benefit across a wide range of threshold probabilities, affirming that our model can effectively guide timely interventions for high-risk individuals while preventing over-treatment in low-risk patients. Given its non-invasive nature, cost-effectiveness, and reliance on routinely accessible clinical indices, this model possesses high feasibility for integration into standard clinical workflows.

## 5 Limitations and future directions

While our nomogram provides a clinically scalable tool for early risk stratification, several limitations merit critical consideration regarding its clinical readiness:

First, merging anxiety and depression into a single composite endpoint introduces clinical and pathophysiological heterogeneity. Although this design optimized statistical power and event rates, anxiety and depression represent distinct clinical entities. In PD, anxiety is frequently linked to locus coeruleus-noradrenergic and amygdala dysfunction, whereas depression primarily involves raphe nuclei-serotonergic pathways and broader subcortical networks. Aggregating these symptoms may mask or dilute the specific predictive efficacy of NfL or clinical scales for individual psychiatric phenotypes, potentially limiting tailored psychopharmacological or targeted psychological interventions.

Second, the retrospective, single-center design poses potential spatial and temporal heterogeneity. Despite rigorous quality control and sample preprocessing, the long enrollment period spanning multiple years may introduce subtle drifts in baseline motor severity. Additionally, potential low-grade degradation from prolonged biobanking and minor inter-batch assay variations across historical samples might have introduced variations in the validation set, explaining the mild, typical reduction in predictive performance compared to the derivation cohort.

Finally, the quantification of plasma NfL exhibits prominent platform- and site-dependence. Discrepancies in assay platforms (e.g., Simoa vs. alternative high-sensitivity immunoassays), pre-analytical protocols, and institutional quality control often cause systematic baseline deviations. Because our model was trained on data from a single center using a specific platform, the absolute cut-off thresholds may experience translation bias if applied directly at other medical centers or with different equipment.

In conclusion, constraints related to a single-center design, composite endpoint definition, and platform-specific biomarker calibration limit the universal generalizability of the current model. Prior to formal clinical adoption as a routine screening instrument, future multi-center, prospective, large-scale studies are urgently needed to facilitate extensive external validation and customized model recalibration. Expanding the sample size will further enable the construction of separate, non-overlapping predictive models for isolated anxiety, isolated depression, and true comorbid anxiety-depression, thereby advancing precision medicine in PD care.

## Data Availability

The raw data supporting the conclusions of this article will be made available by the authors, without undue reservation, upon reasonable request to the corresponding author.
